# Cranioplasty: A Multidisciplinary Approach

**DOI:** 10.3389/fsurg.2022.864385

**Published:** 2022-05-17

**Authors:** H. Mee, F. Anwar, I. Timofeev, N. Owens, K. Grieve, G. Whiting, K. Alexander, K. Kendrick, A. Helmy, P. Hutchinson, A. Kolias

**Affiliations:** ^1^ Division of Rehabilitation Medicine, Department of Clinical Neurosciences, University of Cambridge & Addenbrooke’s Hospital, Cambridge, UK; ^2^ Division of Neurosurgery, Department of Clinical Neurosciences, University of Cambridge & Addenbrooke’s Hospital, Cambridge, UK

**Keywords:** cranioplasty, multidisciplinary approach, rehabilitation, traumatic brain injury, stroke

## Abstract

Decompressive craniectomy (DC) is an operation where a large section of the skull is removed to accommodate brain swelling. Patients who survive will usually require subsequent reconstruction of the skull using either their own bone or an artificial prosthesis, known as cranioplasty. Cranioplasty restores skull integrity but can also improve neurological function. Standard care following DC consists of the performance of cranioplasty several months later as historically, there was a concern that earlier cranioplasty may increase the risk of infection. However, recent systematic reviews have challenged this and have demonstrated that an early cranioplasty (within three months after DC) may enhance neurological recovery. However, patients are often transferred to a rehabilitation unit following their acute index admission and before their cranioplasty. A better understanding of the pathophysiological effects of cranioplasty and the relationship of timing and complications would enable more focused patient tailored rehabilitation programs, thus maximizing the benefit following cranioplasty. This may maximise recovery potential, possibly resulting in improved functional and cognitive gains, enhancement of quality of life and potentially reducing longer-term care needs. This narrative review aims to update multi-disciplinary team regarding cranioplasty, including its history, pathophysiological consequences on recovery, complications, and important clinical considerations both in the acute and rehabilitation settings.

## Introduction

### Background

Decompressive craniectomy (DC) has become increasingly common over the last 15–20 years due mainly to its increased application in traumatic brain injury (TBI) and stroke management (1) either as a primary procedure in the clinical context of expected brain swelling or as a secondary procedure to help treat raised intracranial pressure refractory to medical management, with several clinical trials (2, 3) demonstrating its efficacy. A cranioplasty aims to reconstruct the skull, thus providing cerebral protection and improved cosmesis. It can result in a marked improvement in neurological function due to the physiological effects on the cranial vault, and it should be considered an important step in the rehabilitation process of patients. However, interesting clinical questions remain, including complication rates, neurological recovery and outcomes, the influence timing has on these factors, material choice, and overall cost-effectiveness.

### Historical Aspects

The earliest known neurosurgical operation was trepanning, also known as trephining or trephination, performed in the Neolithic period (4) with skulls found in the graveyards of Paracas and Parachamac in Peru dating back over 3,000 years to prehistorical Inka culture demonstrating this early neurosurgical intervention (5, 6), which is now commonly known as the burr hole procedure. The cranioplasty followed, and ancient authors from Egypt, Greece, and Italy describe its practice. However, it was not until Fallopius (1523–1562), a Flemish physician and anatomist, that the first true cranioplasty was described, who explained that “*the bone could be replaced if the dura was not compromised but a gold cranioplasty considered if the dura was broken*” (7). In 1668, a Dutch surgeon, Job Janszoon van Meekeren, reported the first successful bone graft cranioplasty on a Russian using a canine xenograft. Further advancements included Augustin Belloste (1732), a French surgeon, who described using “*two wings either side of a lead cranioplasty to cover the dura after trephining the skull*” (8). Later, in 1820, a German surgeon, Van Walther, performed the first autologous bone graft for cranioplasty (9). Since then, the surgical technique and the biomaterials deployed in this operation have been improved, and it is now a routine neurosurgical procedure.

### Cranial Reconstruction Multidisciplinary Team

Following a DC, patients often have a multitude of physical, cognitive, functional, and psychological needs, with cranioplasty being an added addition to an often already complex rehabilitation picture. The term “multidisciplinary team” (MDT) refers to activities that involve the efforts of individuals from several disciplines (10), who work cohesively to oversee the care and management of an individual patient. It relies on coherent and effective team work, as no one clinical discipline can solely meet a patient’s needs. The precise composition of the team will vary according to the clinical need and nature of any individual service, but in the context of a cranial reconstruction MDT, core members would include nursing, physiotherapy, occupational therapy, speech and language therapy, dietitians, neuropsychology, neuropsychiatry, neurosurgery, and rehabilitation medicine. The core MDT would oversee cranioplasty logistics, including manufacturing and/or storage and the timing of the operation, as well as overseeing the clinical pathway such as tonal management and neuropsychological issues. In addition, the MDT would aim to have a holistic overview of all aspects of the patient’s rehabilitation, especially ensuring that the cranioplasty is performed for the right indications and at an optimal time with the aim of ensuring the maximal chances of neurological recovery whereever possible. It is important to be able to ask for the advice and assistance of other teams outside the core MDT, and this is termed interdisciplinary team work, with maxillofacial surgery and plastic and reconstructive surgery being two specialties that could be required for complex case discussions from a surgical reconstruction perspective and for orthotists with regard to discussions concerning cranial helmets and other potential protheses.

#### Pathophysiological Mechanisms

The Monroe Kellie doctrine states that “*the sum of volumes of the brain, CSF, and intracranial blood is constant*” (11), and following a brain injury, this equilibrium can be disrupted, often resulting in raised intracranial pressure (ICP), causing a decrease in cerebral perfusion pressure [CPP (mean arterial blood pressure minus ICP)]. Managing this acutely is critical in reducing the chances of brain ischemia, and a primary or secondary decompressive craniectomy may be considered as a management strategy in this context as a lifesaving intervention in those severe cases where there is raised ICP refractory to medical management.

Those who survive will require a second operation a few months later to have their skull reconstructed. This operation, which is termed cranioplasty, is performed for skull integrity, normalization of intracranial physiology, and improvement in cosmesis. Apart from the obvious benefit of restoring a degree of mechanical protection to the brain, it can also improve neurological function (12). The exact pathophysiological mechanisms underlying the possible neurological improvement following CP are not entirely understood, but the improvement is, in part, due to the physiological effects that the restoration of an intact cranial vault has on the brain (13). A negative gradient between atmospheric and intracranial pressure can result in deterioration after craniectomy (14). The cranioplasty stabilizes the atmospheric pressure gradient and re-establishes the fixed volume of the cranial vault allowing the brain parenchyma to re-expand. The disturbance of cerebrospinal fluid circulation and cerebral perfusion is well described in the chronic phase of acquired brain injury as is the improved cerebrospinal fluid hydrodynamics (15–17) and improvements in cerebral blood flow (17–19) following cranioplasty. These pathophysiological consequences are what is proposed to help explain the reasons for possible neurological improvement, but further research is required to understand these principles better (13). Cranioplasty does not have a direct effect on the underlying brain injury, and so neurological outcomes do not always improve following cranioplasty. There is no clear model to date allowing for a prediction of which patients would benefit the most from cranioplasty, and so a better understanding of the pathophysiological consequences and the relationship with neurological outcome remains a priority.

## Clinical Indications

About 65% of decompressive craniectomies are secondary to traumatic brain injuries (TBI) or strokes, with other indications including subarachnoid hemorrhages, tumors, and infections. Most patients who survive will require their skull to be reconstructed by means of cranioplasty, which is often a planned, elective operation. It provides cerebral protection, helps reduce the risk of falls by improving vestibular system equilibrium, as well as recover from the syndrome of trephined. Improved craniofacial cosmesis, although often not the primary purpose of the reconstruction, should not be an overlooked consideration. A growing synthesis of evidence suggests that an early cranioplasty may enhance the chances of neurological recovery, both functionally and cognitively (4, 14, 20). An expediated cranioplasty is sometimes considered, especially in the clinical context of the syndrome of trephined, or in the management and reduction of pulsatile or painful defects post craniectomy. Contraindications for cranioplasty include the presence of any possible infection in the brain or bone and unmanaged hydrocephalus.

### Syndrome of Trephined

First described by Grant and Norcross in 1939 (21) as a syndrome comprising severe headaches, dizziness with pain at the craniectomy site, and an altered cognitive state of mind, the current definition has evolved, comprising three main components (22):

- The occurrence of neurological deficits weeks to months after the craniectomy.
- The occurrence of neurological deficits separates those associated with the initial pathology.
- Clinical resolution after cranioplasty.

As per the definition, the term “syndrome of trephined” should be used only after a cranioplasty has resolved the neurological symptoms, and so before the cranioplasty, the term “sunken flap syndrome” is used; however, in clinical practice, these two terms are interchangeable. It is an underdiagnosed consequence of DC, likely because of the complexity and heterogeneity of outcomes patients suffer following a severe brain injury, and often develops slowly over days or weeks and, in the most severe cases, results in a marked deterioration of the neurological state, leading to coma. It is thought to be due to a negative pressure gradient between the atmosphere and the cranium, which results in neurological compromise (14) due to the sunken flap. Clinical manifestations are variable, with a recent systematic review by Ashayeri et al. (22) showing the most common presenting features to be motor weakness (61%), followed by cognitive deficits (44%), language deficits (30%), altered consciousness (28%), and headaches (20%) with an average of 5 months from craniectomy to symptoms. Fifty-four cases were included in the systematic review, with 34.6% reporting complete recovery from neurological symptoms consistent with SoT following their cranioplasty.

Due to the timing of onset, those patients at most risk are often in rehabilitation facilities, and so an awareness and understanding of this often subtle and difficult clinical scenario is important. Management includes a full clinical assessment to try and ascertain any new or worsening neurological symptoms in addition to those from the underlying brain injury. Positioning of the patient is important, as there is often an improvement in neurology upon lying horizontally, and cranial reconstruction (21, 23) should be expediated.

### Procedure Technique and Considerations

The Guidelines for the Management of Adult Severe TBI (4th Edition) (24) have recommended a minimum size of 12 cm × 15 cm for a frontotemporoparietal DC to reduce mortality and improve neurological outcomes. Cranioplasty surgical planning should be considered early and include the size and location of DC, the need and method for scalp closure, and an identification of the temporalis muscle, all of which will impact the surgical success of cranial reconstruction (25). Ideally, at the time of craniectomy, consideration should be made in relation to maintaining a plane between the brain and the scalp flap. Further surgical considerations include scalp flap elevation, dural soft tissue dissection, and skin flap vasculature. Bifrontal cranioplasty has been shown to increase the risk of complications, with infection being up to 2.5 times higher (26) than hemispheric cranioplasty. In addition, reoperation rates are reported to be higher in patients with bifrontal cranioplasty (27). The location of DC will have an impact on disability profiles, but this relates predominantly to the extent and type of the underlying brain injury. The use of antibiotics at induction and intra-operatively is widely adopted, but the dose and frequency of post operative antibiotics for surgical site infections is not clear.

## Materials

Broad categorization divides materials into either autologous or synthetic. Advancements in materials and manufacturing techniques have led to synthetic materials becoming more common, but the “ideal” or “optimal” material is still unknown. Favorable characteristics include its biogenic compatibility and osteoinductive/osteoconductive properties. The material should be mechanically resistant but easy to manipulate, lending itself to customizable designs and molding. Ideally, it should be able to resist infection and not degrade or conduct heat. No one material ticks all these boxes, which is why such wide-ranging materials are used in clinical practice (**Table 1**).

**Table 1:** Summary of cranioplasty materials.

Material	Implant type	Key points	Considerations
**Autologous bone**	Autograft	Biocompatible Risk of bone resorption	Remains the most used material across the world Relatively low cost, depending on the method of storage/preservation
**Polymethylmethacrylate (PMMA - solid)**	Polymer	Bio-inert No exothermic reaction Easy to contour	Abx incorporation through soaking—beneficial for the management of repeat procedure secondary to infection (22). Widely used Low cost
**Polyetheretherketone (PEEK)**	Polymer	Bio-inert Mechanically resistant (23)	Lack of long-term studies In-house sterilization required
**Titanium**	Metal	Biocompatible Noncorrosive and nonferromagnetic Mechanically resistant	Options for manufacture include plate, mesh, or a 3D porous implant. Associated with better cosmetic and functional outcomes (24) High cost
**Porous Hydroxyapatite (HA)**	Ceramic/polymer	Bioceramic porous material Close biomimetic characteristics of the bone	Customizable Shown to have a positive impact on bone generation and repair

Autologous remains the most frequently used material for cranioplasty globally, but it requires storing and preserving, which is done primarily through two methods: either the material is kept in a subcutaneous abdominal pocket, which first came into vogue in the 1920s, and which has the advantages of providing a sterile environment but requiring further operation/s in the abdomen and a consideration of the associated risks of such or the material is stored in extracorporeal conditions, via cryopreservation in a deep freezer with a temperature of at least −80 degrees celcius. This method was first practiced in the 1950s to reduce the rate of infection, but this has been correlated with devitalization of tissue, with the potential for increased risk of bone resorption. However, a recent systematic review by Corliss et al. (28) found no statistically significant differences in terms of infection and resorption rates while comparing the two methods of storage. Nevertheless, cryopreservation is closely regulated with specific mandated biobanking required in many countries, which severely limits the options for autologous cranioplasty storage and has been one of the driving factors behind synthetic material use.

In addition, improved techniques in medical imaging and biomodeling have further increased cranioplasties, with computer-aided designing and manufacturing (CAD/CAM) being widely used to help in overcoming the shortcomings of intraoperative molding. The 3D-printed cranioplasty is also being increasingly utilized. Analyses comparing existing materials (29–31) demonstrate near equivalence concerning complications, but variable costs also need to be considered (32, 33). Further studies are required to answer the question on the use of ideal material definitively. The greatest barriers to artificial implants include the often high costs and the lack of longitudinal outcome data surrounding their use.

## Timing

**Figure 1:**
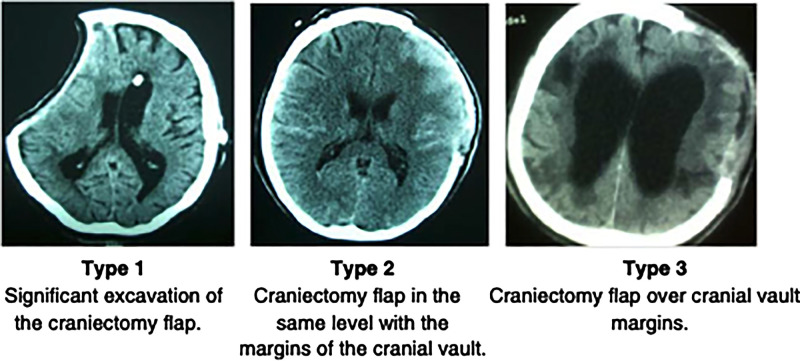
Archavlis et al (34).

Significant debate exists in regard to the optimal timing of cranioplasty and its relationship with postoperative complications and neurological recovery. Broadly, three precranioplasty clinical scenarios (**Figure 1**) (34) exist. After it is determined whether a cranioplasty is clinically appropriate, the debate continues as to the “optimal time.” Delineation of the threshold for “early” and “late” cranioplasty has classically come at, before, and after 12 weeks, but this is continuously challenged. A recent international consensus meeting on post-traumatic cranioplasty suggested that defining a time frame for cranioplasty is somewhat artificial, given the heterogeneity of clinical scenarios, but it can be useful for clinical benchmarking and research purposes (35). Four time frames (post craniectomy) were agreed upon (86.8% agreement): ultra early—up to 6 weeks; early cranioplasty—6 weeks to 3 months; intermediate—3–6 months; delayed—more than 6 months (35). However, variable factors need to be considered, including the patient’s comorbidities, clinical condition such as hemodynamic or respiratory instability, infections, potential wound healing, and progress in the rehabilitation pathway.

In clinical practice, time frames have ranged between 6 and 12 months following DC, as it was thought that an early cranioplasty increased the risk of infection. National hospital episodic statistical data over the past 6–8 years in the United Kingdom show that average timings are reducing in line with recent systematic reviews (31, 36) comparing complication rates with “early” and “late” cranioplasty, which have shown comparable rates of infection across both time frames, challenging the notion that a “late cranioplasty lowers the risk of infection.” A further consideration regarding timing is the procedure length, with a mean time of 109 min for early cranioplasty being significantly shorter (37) than for a late cranioplasty. Although this may not directly affect a patient’s rehabilitation, the timing of the procedure in relation to the rehabilitation pathway may have significant consequences that should be considered. With functional recovery following a brain injury continuing up to at least two years from injury (38) and beyond, it is important to view the cranioplasty as having the potential to enhance the chances of this recovery, and the timing is essential when structuring and planning an inpatient or outpatient rehabilitation program. However, the cranioplasty is often performed in the later stages of an inpatient program or even after discharge, resulting in patients sometimes missing out on the necessary intensive therapy, at a time when the pathophysiological consequences of cranioplasty may be translated into improved neurological outcomes.

## Complications

In most cases, undertaking a cranioplasty is beneficial for a patient, but there is a significant risk profile (30). Overall, complication rates are generally reported at between 15% and 30% (26, 37, 39), with a systematic review in 2016 (36) (18 studies of 3,126 patients) showing an overall pooled complication rate of 19.5% with no difference between time frames.

Common complications include infection, intracranial hemorrhage, extra-axial fluid collections, hydrocephalus, seizures, and bone resorption.

### Infection

Historically, there has been a concern over higher rates of infection in early cranioplasty, but recent studies and systematic reviews have challenged this. In 2016, a multicenter prospective cohort study found no difference in infection rates in early cranioplasty compared with a late one (32). This has been backed up by several more recent SRs (31, 36), with infection rates now commonly accepted as comparably independent of time frames. Infection rates vary between 1.4% and 24.4% (a pooled rate of 7.7%) (36). This range is marked because of what constitutes a definition of infection, which varies from superficial wound infection to subdural empyema requiring cranioplasty removal.

Clinically, indications of infection include scalp tenderness and pain, redness, and swelling, which can be accompanied by surgical scar reopening (40). Severe infections of the cranioplasty resulting in removal are the most concerning and have the greatest impact for a patient. Following removal, reinsertion is often done at least 1 year later, following multiple courses of antibiotics, acute hospital readmissions, and repeated surgeries, all of which can have a significant impact on a patient’s rehabilitation, function, and quality of life.

### Hydrocephalus

Post-traumatic hydrocephalus and subdural hygromas (sub-dural collections of CSF) are common (41), with rates post-cranioplasty ranging between 5% and 45%, dependent on the diagnostic criteria, from the insertion of a VP shunt to asymptomatic ventriculomegaly observed on serial imaging. It can be challenging to ascertain whether hydrocephalus post CP is caused by the cranioplasty and its timing, direct brain injury (13, 37), or the craniectomy. A recent systematic review showed pooled rates of 7.93% (13) independent of the timing with a lower incidence following early CP in the TBI population. It has been postulated that high rates post CP could be related to the size of DC, with large defects resulting in irreversible ventricular enlargement, and following CP, the enlarged ventricles may cause obstruction of the subarachnoid space, increasing resistance to CSF outflow (42). In addition, lower rates of hydrocephalus among those who undergo early cranioplasty are, in part, due to the normalization of ICP dynamics and CSF outflow through improved arachnoid granulation function (43). Delayed CP can result in permanent dysfunction to the arachnoid granulations, leading to persistent CSF outflow dysfunction. One management strategy is the insertion of a ventricular peritoneal (VP) shunt, at the time of cranioplasty, to recover from the effects of post cranioplasty hydrocephalus. Currently, this practice is varied and is usually considered on a case-by-case basis.

Clinically, regardless of cause, hydrocephalus poses diagnostic challenges both in the acute and in the rehabilitation settings. It should be on the radar of any clinician caring for patients following DC, whether they are pre or post CP. Active imaging and management should be considered in those patients who are slow to wake, making poor progress, or neurologically deteriorating (13, 37). If enlarged ventricles are apparent radiologically, then referral to neurosurgery should be considered, which will often result in imaging, and if necessary, infusion studies should be conducted and the insertion of a VP shunt should be considered for comprehensive management.

### Seizures

The estimated incidence of seizures following cranioplasty is 6.1% (36), but it can be difficult to delineate this risk secondary to cranioplasty compared with the risks from the underlying brain injury. Risk factors include TBI, hemorrhagic stroke, postoperative infection and hemorrhage, male sex, and neurological deficits pre cranioplasty (44). Delineation can also be difficult, given that the numbers of cofounding factors are precise about the effect of an antiepileptic drug (AED) prophylaxis on seizure rates post cranioplasty, but studies have shown a significant reduction in the rates of seizures post cranioplasty if an AED is prescribed for at least seven days (45, 46). In practice, patients are often already on an AED at the time of cranioplasty because of the underlying brain injury.

### Bone Resorption (BFR)

Bone flap resorption is a well-recognized complication following autologous cranioplasty and one that can have a significant impact for patients, especially in terms of requiring further surgery for removal and/or revisions and cosmetic outcome. The pathophysiology of BFR is not entirely known but is likely due to variable scalp and dural blood supplies and the lack of incorporation to the surrounding bone, with infection being a likely contribution. Other risk factors include multiple fractures, bone fragmentation, larger cranioplasty sizes, younger ages, ventriculoperitoneal shunts, and early cranioplasty (32). Bone flap resorption may result in a more substantial defect, resulting in further complications and surgeries, and potentially a deterioration in the cosmetic appearance of the patient.

A topic discussed at an international consensus meeting on post-traumatic cranioplasty (35) related to BFR, with 100% agreement that bone graft carries a risk, but with 88.5% consensus on the unclear role of storage methods (page 6) in resorption rates.

## Rehabilitation Considerations

### Integrated Rehabilitation MDT for Cranioplasty

Patients following decompressive craniectomy often require complex disability management as described above, and structured periods of inpatient rehabilitation are common. In the United Kingdom, this would be often undertaken in a combination of level 1 and level 2 rehabilitation units by an specialised MDT. There is a heterogeneity of outcomes for patients following DC, ranging from those in a prolonged disorder of consciousness through to those regaining functional independence. Cranioplasty should be a consideration for all categories of patients, but perhaps for different reasons. The structure of a rehabilitation program should be set within a conceptual health framework such as the WHO’s “International Classification of Functioning, Disability and Health (ICF) (47) to ensure that the multiple facets of the cranioplasty are considered. The cranioplasty must be performed only “if” and “when” clinically appropriate. Ideally, these decisions should be made as part of a cranial reconstruction MDT, with consideration given to the parameters outlined in this review, hopefully resulting in optimal care for an individual patient and allowing for maximal potential improvement in neurological outcome.

### Positioning and Mobility

Patients recovering from a TBI or stroke often have a complex mix of physical, cognitive, psychological, and psychosocial needs. These can vary hugely, with no one case being the same, but a skull defect adds an extra layer of complexity to the rehabilitation picture. Difficulties with positioning, mobilization, and general personal care are common. Patients with a sunken flap often present with increased tone, requiring a 24-hour postural management plan, a consideration of orthotics and splints, to optimize the position of the head, pelvis, trunk, and limbs, alongside medical management through the utilization of global and focal antispasmodics.

The risk of falls for patients following a brain injury is always a concern for the MDT, exacerbated following a craniectomy due to physical impairments, increased impulsive behaviors, cognitive impairments, and disequilibrium. The safety of patients is always paramount, and a survey showed that the confidence of physiotherapists mobilizing stroke patients post hemicraniectomy was lower than for those without a skull defect (48), demonstrating an understandable, more cautious approach with this cohort of patients. There may well be reduced higher-level balance or cardiovascular training for this cohort, and as there is a lack of guidance, a more precautionary approach may be adopted. This approach is likely to be similar across the MDT, although there is limited evidence for this, potentially resulting in differing patterns of rehabilitation interventions for these patients, which could be detrimental to them. The cranioplasty reduces the risk of falls through varying physiological effects and is likely to give the rehabilitation MDT an added confidence to approach and treat these patients.

The use of cranial helmets in this setting is varied, especially in the adult population. Helmets are still commonly prescribed following pediatric craniectomies but only in individual cases for adults who pose a particular risk. There is no objective evidence showing that patients sustain increased head injuries because of trauma between a craniectomy and a cranioplasty. This may well be because this cohort of patients is less likely to be in a position of sustaining a severe head injury, and if they are mobilizing or socially active, this will often be with a therapist, career, or family member, and so the risk is likely to be reduced.

### Cosmesis

Craniofacial cosmesis can often be difficult to objectively measure as it is so subjective to an individual patient. However, before the cranial reconstruction, patients may be reluctant to engage in inpatient or outpatient settings due to cosmetic appearance and confidence. This has the potential to impact their rehabilitation path, self-esteem, and mental health. Some cranioplasty cosmesis scales are available following cranioplasty. However, they are not widely utilized in clinical practice to date, and often clinicians use a visual analogue scale to record cosmetic acceptance, but there is very little evidence showing how this may impact a patient’s rehabilitation.

### Driving

The main limiting factor for return to driving following cranioplasty is with the risk of seizures, which are reported to be around 6% following cranioplasty (36). There may be other reasons why driving may not be appropriately linked to the underlying brain injury, but the cranioplasty itself should not limit driving in the future. The usual pathways should be followed for a person to return to driving. In the United Kingdom, the patient needs to inform the DVLA that they have had a cranioplasty, and if applying for a group 1 license, this is not an absolute contraindication to driving, but the underlying condition leading to surgery will need to be considered before a license can be returned.

### Contact Sport

This is very much on a case-by-case basis, and at the advice of the neurosurgical team and a wider MDT, and depends on the type of contact sport. With synthetic materials often having a greater tensile strength than autologous bone, the cranioplasty itself is often not the limiting factor when considering a return to contact sport but instead the underlying brain injury.

### Flying

The Civil Aviation Authority (CAA) recommends at least 7 days from neurosurgical intervention to flying due to the risk of pneumocephalus expanding at altitude, but it does not give specifics to more complex interventions, and so, each case should be reviewed by an appropriate specialist. However, in regard to cranioplasty, once a patient has recovered fully from the operation, then there are no specific restrictions to flying but, again, a consideration of the underlying brain injury should be reviewed. Flying between decompressive craniectomy and cranioplasty is a much more complex proposition, and although there are no specific guidelines for this, whenever possible, it should be undertaken with the appropriate clinical expertise.

## Outcomes Following Cranioplasty

### Neurological Outcome

Quantifying and categorizing neurological change is a challenge, especially across a cohort with such heterogeneity of clinical baselines, from those in a prolonged disorder of consciousness, through to those on a trajectory to a good functional outcome independent of cranioplasty. There are multiple descriptions of neurological improvement following cranioplasty (**Table 2**), broadly divided into physical, functional, and cognitive outcomes. However, given the multiple confounding factors, including severity of the injury, craniectomy location, timing, and intensity of rehabilitation, there is “no one answer fits all,” and it is essential to approach each case individually. A 2018 systematic review explored the motor and cognitive changes following cranioplasty and showed that procedures performed within 90 days improved motor function, whereas the Mini-Mental State Examination (MMSE) or memory function did not significantly alter (49). However, a further systematic review comparing “early and late” cohorts showed no difference in neurological baseline pre cranioplasty but significantly improved outcomes in the early cohort (12). Further case series show a speeding up of the process of functional, physical, and cognitive recovery by early cranial reconstruction (4, 20, 34), which lends credence to the notion that “earlier is better,” but there is no definitive evidence or consensus on the optimal timing for cranioplasty; further prospective studies are required to define time frames.

**Table 2:** Influence of timing on complications and neurological outcomes—recent systematic reviews.

Author/year	Title	Results	Conclusion
Malcolm et al. (36)	*Complications following cranioplasty and relationship with timing: A systematic review and meta-analysis*	Total of 3,126 patients (1,421 early vs. 1,705 late). Early CP had significantly higher odds of hydrocephalus than late CP. There is no difference in overall complications, infections, re-operations, intracranial hemorrhage, extra-axial fluid collections, seizures, or bone resorption.	Early CP within 90 days after DC is associated with increased odds of hydrocephalus than with later CP, but no difference in the odds of developing other complications.
Malcolm et al. (12)	*Early Cranioplasty is Associated with Greater Neurological Improvement: A Systematic Review and Meta-Analysis*	Total of 528 patients. CP, regardless of timing, was associated with significant neurological improvement. Neurological outcome was significantly improved in the early cohort and showed a greater magnitude of change than late CP.	CP may improve neurological function, and an early CP may enhance this effect. Future prospective studies evaluating long-term neurological outcomes are required.
De Cola et al. (49)	*Timing for cranioplasty to improve neurological outcome: A systematic review*	Total of 162 patients. Early CP (<90 days) is more effective in improving motor functions, but it does not significantly improve the MMSE score or memory functions.	CP performed from 3 to 6 months after DC may significantly improve both motor and cognitive recovery.

It is also important to remember that there is no certainty of neurological improvement following cranioplasty. There is a suggestion that cranioplasty has the greatest effect on neurological recovery in those patients who were neurologically improving independent of cranioplasty, with the cranioplasty helping to optimize the pathophysiological state of the cranial vault as discussed, resulting in the most optimal state for neuroplasticity and neurological improvement. However, further work is required to understand which patients would benefit the most. Largely subjectively observed in clinical practice, because the evidence is lacking, there are also more subtle areas of change post cranioplasty that can have a great impact on a patient’s rehabilitation potential, quality of life, and overall outcome, including positioning, tone, and spasticity.

### Impact of Cranioplasty on Cognition

Cognition is a key part of neurological recovery that can have a large impact on functional independence, quality of life, and general wellbeing. The role that cranioplasty can play in cognitive change does need to be cautiously evaluated as there are many confounding factors that need to be taken into account, but an increasing number of reviews and case series have demonstrated a significant improvement in cognition following CP (20, 49–52). Cognition itself is an umbrella term, and an ideal testing plan should include the five domains of cognitive function, namely, memory, language, attention, visuospatial functioning, and executive function. This is often performed as part of a cognitive battery of tests but not always reported as such.

One case series by Di Stefano et al. (20) showed a greater cognitive change in patients who had a cranioplasty within 6 months from DC, and, therefore, it was recommended that cranioplasty be considered a key factor in the neuropsychological recovery and should be performed at the earliest opportunity to take advantage of the optimal window for rehabilitation. However, a 5-year retrospective study by Corallo et al. (50) again demonstrated cognitive improvement post CP but with the most significant differences after 4 years post CP.

It is important to remember that not all patients have cognitive improvements post cranioplasty, and it is hard to separate improvement that would be expected over the natural time frame from DC compared with direct improvement as a result of CP, but these issues should at least be discussed and considered as part of the patient’s rehabilitation. As discussed earlier, a better understanding of the factors that predict neurological recovery post CP should be a focus of future research.

## Future Research Directions

### Future research directions

- Timing of cranioplasty and its relationship with neurological outcome
- The relationship of cranioplasty with intracerebral fluid hydrodynamics and clinical recovery
- Optimal material choice for cranial reconstruction
- Long-term outcomes following cranial reconstruction

## Conclusion

Cranioplasty is a well-practiced and utilized neurosurgical procedure aiding in skull reconstruction. It has benefited from improvements in technology, operating techniques, and manufacturing methods over the past 10–15 years. No one clinical scenario is the same, but ideally the cranioplasty should be discussed and considered by an MDT for patients following a craniectomy. In patients where it is considered, complications, timing, quality of life, and cosmesis are all factors that should be addressed, and a good dialogue between the surgeon and the rehabilitation team is essential to maximize recovery and improve outcomes. With these decisions and with planning being incorporated into a framework such as the international classification of functioning disability and health, cranioplasty can be utilized as a vital ingredient of a patient’s rehabilitation program following a brain injury.
